# Omega fatty acids and resolution of inflammation: A new twist in an old tale

**DOI:** 10.4103/0972-124X.65426

**Published:** 2010

**Authors:** Antush Mittal, V. Ranganath, Ashish Nichani

**Affiliations:** *Department of Periodontics, AECS Maruti College of Dental Sciences and Research Centre, Bangalore ‐ 575 076, India*

**Keywords:** Lipoxins, omega fatty acids, protectins, resolution of inflammation, resolvins

## Abstract

Identification of the factors that regulate immune tolerance and control the appearance of exacerbated inflammatory conditions is crucial for the development of new therapies of inflammatory and autoimmune diseases. Resolution of inflammation and the return of tissues to homeostasis protect us against excessive tissue injury and promote the restoration of function and structure. Resolution of inflammation, which was considered a passive event, is actually an active process where new families of endogenous lipid mediators from omega-3 polyunsaturated fatty acids play an important role in removing proinflammatory mediators generated from arachidonic acid. These chemical mediator families, termed *Resolvins* and *Protectins*, are potent stereoselective agonists that control the duration and magnitude of inflammation, along with the *Lipoxins* as signals in resolution. This review examines the mapping of these circuits and recent advances in our understanding of the biosynthesis and actions of these novel proresolving lipid mediators. A search in the electronical databases PubMed and the Cochrane Central Register of Controlled Trials was carried out. The search strategy applied was: “Omega fatty acid” AND “resolution of inflammation,” including articles from January 1,1985 to October 2009. This resulted in the identification of a total of 52 articles, which were analyzed in full text leading to consideration of only nine full texts.

## INTRODUCTION

The inflammatory response is, in general, protective, and ultimately rids tissues of both the cause and the consequences of tissue injury that can accompany host defence. Acute inflammation, defined by its cardinal signs: *dolor, calor*, and *rubor*, may lead to chronic inflammation, scarring, and eventual loss of function if the tissue fails to completely resolve the inflammation.[1]

The vascular and cellular responses of both acute and chronic inflammation are mediated by endogenous chemical factors derived from plasma or cells and triggered by the inflammatory stimulus.[2]

Such mediators, acting alone, in combination, or in tandem, then amplify the inflammatory response and influence its evolution and the outcome of the process.[2]

This review gives an overview of the novel potent lipid mediators that have been identified in recent years within resolving exudates that demonstrate potent bioactions. The first family of mediators recognized with anti-inflammatory and proresolving properties is the lipoxins, generated form arachidonic acid (AA). In contained sites of inflammation, lipoxins are temporally dissociated from other proinflammatory mediators such as the prostaglandins and leukotrienes that are biosynthesized in the initial steps of the acute inflammatory response. The two new families of compounds identified within the resolution phase were termed resolvins (Rv) and protectins (PD) because of their unique chemical structures and in recognition of their potent stereoselective bioactions. The isolation, structural elucidation, and characterization of these novel endogenous mediators that are actively biosynthesized within the resolution phase of acute inflammation established that resolution is an active process rather than a passive process, as once widely believed. Moreover, they provide clear evidence that the resolution of acute inflammation is preprogrammed and tightly controlled at the tissue level to proceed in a nonphlogistic fashion.[1]

This review focuses on the proresolving mediators that are generated from essential polyunsaturated fatty acids (PUFAs) – AA, eicosapentaenoic acid (EPA), and docosahexaenoic acid (DHA), namely lipoxin, Rv, and PD – and on their biosynthetic pathways and action.

## RESOLUTION PHASE OF ACUTE INFLAMMATION

**Figure 1:**
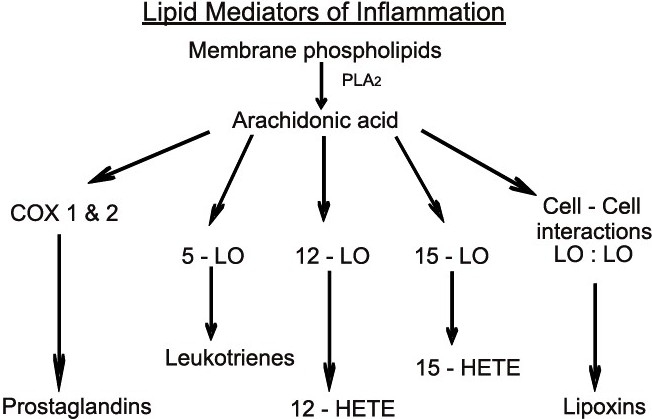
Endogenous production of lipid mediator using omega-6 fatty acid, i.e., arachidonic acid, which is cleaved by phospholipase A2 (PLA2) and becomes the substrate for cyclooxygenases (Cox-1 and -2) and Lipooxygenases (5-LO, 12-LO, and 15-LO) to form potent proinflammatory mediators. Later, with specific transcellular and cell:cell-specific interactions, they form lipoxins, which help in resolution. Thus, active production of endogenous lipid meditors help in the resolution of inflammation

On initiation of an inflammatory event (either by injury or by microbial challenge), the prompt destruction of the deployed leukocytes requires that the tissues return to homeostasis or complete resolution without leaving molecular signs or traces of the host leukocyte battle, for example with invading microbes, at the tissue level. Complete resolution is the ideal outcome for the tissue where leukocyte infiltration, phagocytosis of cellular debris, and/or destruction of invading organisms is in progress[3] [Figure 1].

## ANTI-INFLAMMATION VERSUS RESOLUTION

Anti-inflammation can block or inhibit both exogenous and endogenous proinflammatory chemical mediators, but it is not equivalent to proresolution. Cyclooxygenase (COX)-inhibitors prevent prostaglandin biosynthesis and are effective anti-inflammatory mediators; however, they prolong the time to resolve and thus extend tissue inflammation. But, for proresolving, it must be an agonist to resolution via targeting cell types and events critical for the tissue to return to homeostasis.[1]

## CHEMICAL MEDIATORS IN INFLAMMATION AND RESOLUTION

**Figure 2:**
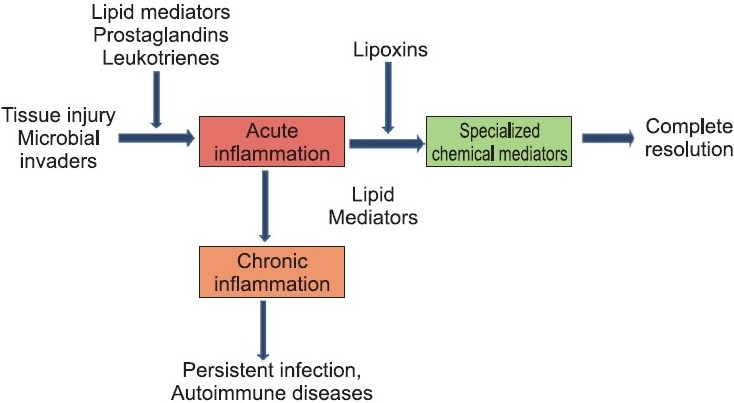
Various outcomes of acute inflammation

In health status, inflammatory responses are self-limited, with many cell types and tissues involved in initiation and termination of acute inflammation. The first level of control of this cellular traffic involves the generation of local endogenous signals or autacoids that drive the host’s response. These chemical mediators or signals are local-acting and mediate the visible signs of inflammation[1] [Figure 2].

Prostaglandins were isolated and the term was coined in 1930 by U. von Euler. Leukotrienes were isolated by Samuelsson and colleagues in the late 1970s, their discovery giving a new knowledge to the mechanism of the inflammatory process. All the lipid mediators identified earlier, including prostaglandins and leukotrienes derived from AA as well as related lipid-derived substances such as platelet activating factor, are noted as proinflammatory mediators because they evoke the signs of inflammation.[2]

Lipid-derived mediators are well positioned to play key role(s) as signalling molecules in inflammation because they are small molecules, local-acting, rapidly generated, and locally inactivated. Pro-inflammatory prostaglandins and leukotriene B4 (LTB4) control local blood flow, vascular dilation, and permeability changes needed at the site for leukocyte adhesion, diapedesis, and recruitment.

Both prostaglandin PGE2 and PGD2 also signal the end of the inflammatory process by activating the transcriptional regulation of 15-LOX in human neutrophils, which in turn gives rise to the temporal dissociation of eicosanoids and production of LXs.[4]

The essential roles of omega-3 PUFAs in health were evident in 1929. Omega-3 PUFAs are widely held to act via several possible mechanisms, such as preventing conversion of arachidonate to proinflammatory eicosanoids or serving as an alternative substrate producing less-potent products.[5,6]

Proresolving lipid mediators are a novel genus of endogenous chemical mediators, including lipoxin, Rv, and PD, which are involved in acute inflammation. They are actively biosynthesized in the resolution phase of acute inflammation and are potent agonists that control the duration and magnitude of inflammation [Figure 2]. They are also potent chemoattractants, but act via a noninflammatory mechanism: e.g., lipoxins activate mononuclear cell infiltration without stimulating the release of proinflammatory chemokines or activation of proinflammatory gene pathways and products. They also stimulate the uptake of apoptotic Polymorphonuclear neutrophils (PMNs) (PMNs, the first line of host defence) and activate endogenous antimicrobial defense mechanisms as well as clearance on mucosal surfaces. Together, these dual actions are agonistic; via acting on separate cell populations, they stimulate resolution of inflammation.

Lipoxins are generated during the resolution of acute inflammation to serve in healthy termination. Importantly, these specialized chemical mediators serve as agonists for endogenous anti-inflammatory and proresolving mechanisms.[7]

**Figure 3:**
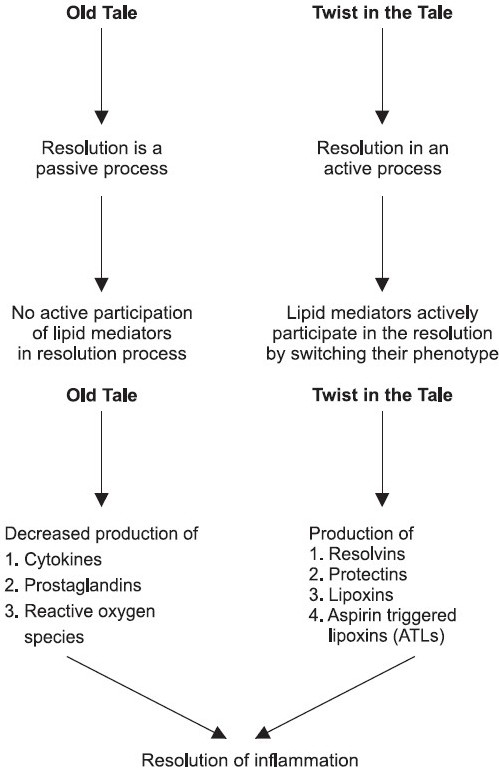
Omega fatty acids and resolution of inflammation: a new twist in an old tale

Resolution phase is composed of active biochemical processes that turn on specific proresolution biochemical signalling circuits [Figure 2]. These concepts came with the identification of specialized chemical mediators biosynthesized during the resolution phase, such as the *Rv* that actively promote resolution and the return to homeostasis[3] [Figure 3].

## CHEMICAL MEDIATORS THAT HELP IN RESOLUTION

1. Lipoxins
2. Aspirin-triggered lipoxins
3. Rv
4. PD

### Lipoxins

**Figure 4:**
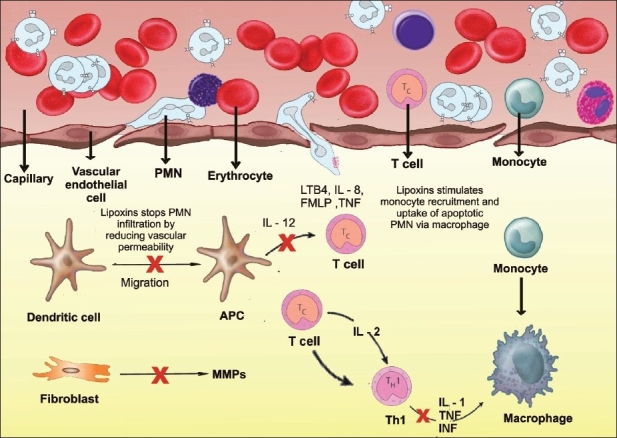
Lipoxins in resolution: dual actions on PMNs’ and monocytes’ stop-and-go signals. Initial chemoattractants recruit PMNs that diapedese from postcapillary venules, an event that is amplified by the production of a 5-LO-pathway product, LTB4, a potent chemoattractant. During progression of inflammatory events, platelet–leukocyte interactions stimulate the formation of lipoxin A4 (LXA4) and LXB4, which serve as stop signals, illustrated by a red x, by blocking the further recruitment of PMNs from the postcapillary venules. This strategic location limits the number of neutrophils required to combat microbes and/or to clean up tissue debris. Lipoxins also stimulate nonphlogistic recruitment of monocytes and stimulate the uptake of apoptotic PMN by macrophages. Lipoxins reduce dendritic cell motility and reduce IL-12 and also regulate T cell cytokines and Matrix metallo proteinases (MMPs) from the fibroblasts, which lead to anti-inflammation and proresolution

The lipoxin series are trihydroxytetraene-containing bioactive eicosanoids first isolated from human leukocytes. The name lipoxins was introduced because they are lipoxygenase (LO) interaction products with distinct structure and actions that are unique among eicosanoids. Lipoxin A4 (LXA4) is 5S,6R,15S-trihydroxy-7,9,13-*trans*-11-*cis*-eicosatetraenoic acid and lipoxin B4 (LXB4) is its positional isomer 5*S*, 14*R*,15*S*-trihydroxy-6, 10, 12-*trans*-8-cis-eicosatetraenoic acid. Lipoxins were the first mediators recognized to possess specific proresolution actions. They are potent stop signals for PMN infiltration, limiting their recruitment to sites of inflammation. Lipoxins stimulate the return of vascular permeability to homeostasis. Lipoxins are dual-acting players and stimulate the nonphlogistic recruitment of mononuclear cells[1,8] [Figure 4].

The process of transcellular biosynthesis is defined as the generation of new bioactive compounds that neither cell type can produce on its own. For example, human platelets do not produce LX on their own. When platelets adhere to PMNs, the platelet-PMN aggregates become a major intravascular source of LX that in turn halts further PMN diapedesis and recruitment.[1,6,8]

#### Mucosal and vascular cell–cell interactions

The lipoxin biosynthetic circuit in cells enriched in 15-LO initiates the oxygenation of AA by 15-LO to generate 15S-H (p) ETE (hydroperoxyeicosatetraenoic acid) transformed into an epoxytetraene intermediate generated by leukocyte 5-LO and specifically open to LXA4 and LXB4.[5,6]

#### Platelet–leukocyte interactions

Platelets adhering to neutrophils can pick up and transform released 5-LO intermediate LTA4 and convert this to LXA4 and LXB4 by platelet 12-LO.[5,6]

#### Membrane phospholipid priming, stored precursors of 15-HETE

Membrane precursors other than AA could be released and converted to bioactive substances. 15-HETE is stored in membrane inositol-containing lipids that are then charged and/or primed to produce lipoxins on activation of cells by releasing the stored 15-HETE that is taken up and transformed by the neighbouring leukocyte. This also leads to concomitant reciprocal reduction in leukotriene biosynthesis and to a dynamic reciprocal relationship between leukotrienes and lipoxin biosynthesis.[1,3,6]

### Aspirin-triggered lipid mediators

Aspirin impacts lipoxin generation. Aspirin triggers the formation of the 15R-epimers of the lipoxins, specifically 15-epi-LXA4 and 15-epi-LXB4. These epimers carry their carbon-15 alcohol group in the *R* configuration. Thus, along with inhibiting prostaglandin formation, which is the well-appreciated mechanism of action, aspirin also triggers biosynthesis of local lipid mediators. The aspirin-triggered epimers of lipoxins are longer acting because they show reduced catalytic activity for enzyme inactivation.[4,8]

15-epi-LXA4 acts at the same receptor as LXA4, i.e. ALX/FPRL1. This receptor shows unique signalling properties and rapidly regulates the phosphorylation of leukocyte-specific proteins in neutrophils, which in turn puts the brakes on PMN migration. This is an exciting new treatment approach and activation of this receptor by nonlipoxin peptides that are also generated endogenously from glucocorticoid treatment such as annexin-1 peptide makes this an interesting and perhaps important control point in endogenous anti-inflammation and resolution.[3]

### Rv and PD

The bioactive local mediators, or autacoids, that require enzymatic generation from the omega-3 essential fatty acid EPA resolving inflammatory exudates *in vivo* and carry potent stereoselective biological actions. They were termed resolvins of the E (RvE) series derived from EPA. Those derived from DHA were termed Rv of the D series. The other family of bioactive chemical signals from DHA (i.e., docosanoids, oxygenated products from DHA), which specifically possess a conjugated triene double-bond system in their structures, are denoted protectins. The protectins demonstrate anti-inflammatory and neuroprotective actions in vivo. Oxygenated compounds identified earlier from omega-3 PUFAs such as prostaglandins and leukotrienes [i.e., leukotriene B5 (LTB5)] were found to be far less potent than their AA-derived counterparts, or completely devoid of bioactivity. Rv and PD evoke biological actions in the nanogram range *in vivo* and are natural exudate products.[3,7]

The term Rv (resolution-phase interaction products) was first introduced to signify that these new structures were endogenous mediators, biosynthesized in the resolution phase of inflammatory exudates, possessing very potent anti-inflammatory and immunoregulatory actions.[1]

These actions include reducing neutrophil traffic, regulating cytokine and reactive oxygen species, and lowering the magnitude of the response. The term neuroprotectin is applied to protectin that is generated in the neural tissues and has anti-inflammatory properties.[3]

These mediators are considered to play a key role in many prevalent diseases not previously considered to be of an inflammatory etiology. These include Alzheimer’s disease, cardiovascular disease, and cancer, along with arthritis and periodontal disease.[1,7]

## LIPID MEDIATORS CLASS SWITCHING IN RESOLUTION (AT THE TISSUE LEVEL)

**Figure 5:**
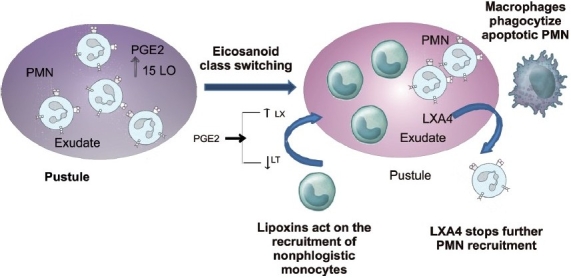
Eicosanoid class switching from proinflammatory mediators to anti-inflammatory mediators that help in resolution

A key event in resolution is the temporal switch in the lipid-mediator class from pro- to anti-inflammatory eicosanoids, which has direct implications for the treatment of inflammatory diseases. Signaling pathways leading to PGE2 and PGD2 in turn actively switch on the production of enzymes required for the generation of lipoxins in human PMN (Neurophils) along with the generation of prostaglandins and leukotrines in the early stages of inflammation to govern vasodilatation and diapedesis respectively.[2] This temporal switch in lipid mediator class is an active process that underscores the ability of leukocytes themselves to trigger the self-limited response of acute inflammation [Figure 5].

The lipoxins are appreciated for their ability to actively promote resolution by regulating the entry of new neutrophils to sites of inflammation and organs of reperfusion injury. They reduce vascular permeability while also stimulating the nonphlogistic infiltration of monocytes that appears to be required for wound healing. They also stimulate macrophages to uptake apoptotic neutrophils.[3,4]

## 5-LO (LIPOOXYGENASE)AND THE NEUTROPHIL SWITCH PHENOTYPE

The neutrophil’s switch in mediators from leukotriene to lipoxin indicates that the profile of mediators formed is not only substrate-dependent from the local microenvironment of the exudate but also 5-LO dependent. 5-LO plays a critical role in exudates, generating proinflammatory mediators initially in recruitment, i.e., LTB4, and later phase mediators lipoxins and Rv. Thus, inhibition of the 5-LO may not be useful in treating all forms of inflammatory diseases. AA-derived mediators temporally turn next to the conversion of omega-3 precursors in exudates to generate Rv and PD, and are also an example of switch phenotype.[3]

## SUMMARY

### Active biosynthesis in resolution

The new families of EPA- and DHA-derived chemical mediators reviewed here, namely the Rv and PD, as well as the arachidonate-derived lipoxins, now open new avenues for designing “resolution targeted” therapies to control the unwanted side of aberrant inflammation. Aspirin appears to have a unique property among anti-inflammatory drugs in that aspirin jump starts resolution by generating epimers of resolution mediators. PUFA-derived local mediators in the resolution of tissue catabasis, i.e., the tissue’s return from battle to homeostasis, which are lipoxins, Rv, and PD. They provide evidence for endogenous anti-inflammatory and proresolving mechanism(s) that are likely to account in part for some of the immunoregulatory and disease-modifying actions attributed to dietary omega-3 PUFAs. The identification of anti-inflammatory and antifibrotic actions of the lipoxins and of aspirin-triggered lipid mediators (ATLs) break the prevailing view of some that all of the many AA-derived mediators are solely proinflammatory as well as pivotal roles of the PMN 5-LO in producing both proinflammatory leukotrienes as well as lipoxins and Rv.[1,4]

Omega-3 PUFAs as endogenous precursors of chemical mediators in the resolution phase are termed Rv and PD. These novel compounds, derived from EPA and DHA, carry anti-inflammatory properties. Hence, Rv and PD might link at the tissue and molecular level, some of the beneficial actions attributed to dietary omega-3 PUFAs.[1]
